# Receptor-Mediated Drug Delivery: Redefining Targeted Drug Conjugates in Oncology

**DOI:** 10.3390/pharmaceutics18030386

**Published:** 2026-03-20

**Authors:** Keon Niles Jafari, Charlene Chai, Shelby Kim, Kamaljit Kaur

**Affiliations:** 1 Department of Biomedical and Pharmaceutical Sciences, School of Pharmacy, Harry and Diane Rinker Health Science Campus, Chapman University, Irvine, CA 92618, USA; 2 Center for Targeted Drug Delivery (CTDD), School of Pharmacy, Chapman University, Irvine, CA 92618, USA; 3 Schmid College of Science and Technology, Chapman University, Orange, CA 92866, USA

**Keywords:** targeted drug delivery, cell surface receptors, overexpression, drug conjugates, tumor drug concentration

## Abstract

Targeted drug delivery (TDD), specifically through targeting ligand–drug conjugates, has reshaped oncology by enabling selective delivery of cytotoxic payloads to cancer cells while minimizing uptake by normal tissues. A key approach relies on exploiting overexpressed cell surface receptors (CSRs) to enable selective uptake of drug conjugates via receptor-mediated endocytosis. This review delineates four clinically validated CSRs (HER2, Trop-2, Nectin-4, and SSTR2) with several FDA-approved drug conjugates. Furthermore, emerging CSRs (EGFR, DLL3, and keratin 1) that may support next-generation TDD platforms for cancer treatment are also highlighted. We discuss how CSR type, density on cancer cells, and its mechanism of endocytosis, as well as the conjugate design for cellular uptake, tissue distribution, ligand size, and linker stability, collectively determine tumor drug accumulation and therapeutic efficacy. From representative examples, we elucidate the rationale for judicious refinement of these parameters, guiding the development of more potent ligands and drug conjugates to enhance the therapeutic efficacy of cytotoxic agents.

## 1. Introduction

**Figure 1:**
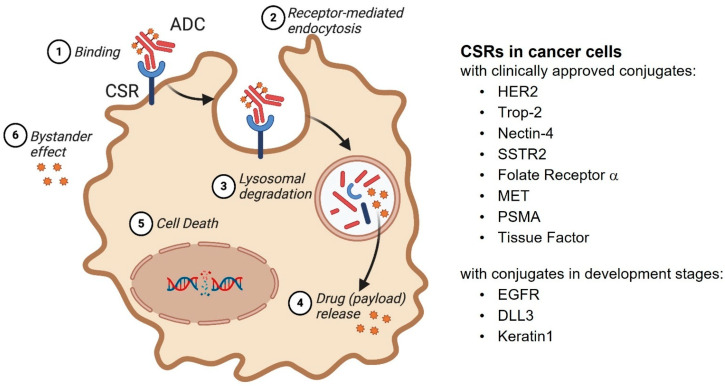
Utilization of cell surface receptors (CSRs) for specific uptake of drugs in cancer cells. CSRs facilitate trafficking of ligand–drug conjugates, such as ADCs and PDCs, via receptor-mediated endocytosis into cancer cells, where the free drug is released inside the cells to impart activity. Additionally, the free drug can also be released to the extracellular tumor microenvironment (TME) for bystander effect to kill nearby cancer cells.

Targeted drug delivery (TDD) has transformed the landscape of oncology by enabling more precise delivery of cytotoxic payloads to malignant cells while minimizing damage to normal tissues. A key strategy in TDD is to exploit overexpressed cell surface receptors (CSRs) on cancer cells to drive selective uptake of drug conjugates through receptor-mediated endocytosis. This receptor-based targeting emphasizes many conjugate therapies, including antibody–drug conjugates (ADCs) [1,2,3,4] peptide–radionuclide conjugates (PRCs) [5,6] and peptide–drug conjugates (PDCs) [7,8,9], enabling the selective delivery of high payload concentrations to the tumor (Figure 1). Notably, several receptors, including HER2, Trop-2, and Nectin-4, have emerged as clinically validated targets [3] with corresponding FDA-approved conjugates now integrated into the treatment regimens for various malignancies (Table 1).

The CSR-directed drug conjugate approach involves linking cytotoxic agents to targeting ligands that specifically recognize and bind to CSRs overexpressed on cancer cells. This strategy typically comprises four key components. First, the identification and selection of an appropriate CSR are critical, as receptor overexpression must be sufficiently high (≥3-fold compared to normal tissues) and cancer-specific to ensure selective targeting [10]. Second, a ligand—such as a monoclonal antibody (mAb), engineered protein, or peptide—with high affinity and specificity for the chosen receptor is required to mediate targeted delivery. Third, a highly potent cytotoxic payload, such as a chemotherapeutic agent, is selected to effectively kill cancer cells upon internalization. Finally, the linker that covalently conjugates the targeting ligand with the payload plays a crucial role. The linker must ensure systemic stability to prevent premature drug release in circulation while allowing controlled release at the tumor site, often through tumor-selective linkers, such as enzyme cleavable tetrapeptide Gly-Gly-Phe-Gly, dipeptide Val-Cit, or acid-sensitive linkers (e.g., carbonate or hydrazone) [7,11]. The bystander effect, which allows free drug released after linker cleavage to persist in the tumor microenvironment, also contributes to killing neighboring cancer cells that may lack the CSR through uptake driven by diffusion. However, CSR-mediated internalization is the predominant pathway for drug accumulation in tumor cells.

In this perspective review, we focus on four clinically validated CSRs, namely, HER2, Trop-2, Nectin-4, and somatostatin receptor (SSTR2), for which FDA-approved conjugates already exist. These receptors serve as representative case studies through which we examine how receptor biology influences the performance of targeted drug delivery (TDD) systems. We also highlight three additional emerging targets: EGFR, DLL3, and keratin 1, which illustrate how next-generation CSRs are being explored for TDD conjugates. Rather than providing an exhaustive catalog of receptors or conjugate formats, we use these representative examples to emphasize how key receptor properties, including surface abundance, tumor selectivity, internalization kinetics, and trafficking pathways, can strongly influence conjugate efficacy and treatment outcomes.

Using these receptors as a framework, we examine how mAbs or peptide ligands can be used to selectively deliver cytotoxic drugs and other therapeutic payloads to cancer cells while minimizing exposure to normal tissues. In doing so, we also highlight broader design considerations that emerge across different TDD platforms, including how targeting ligand format, linker chemistry, and payload class can influence pharmacokinetics, tumor penetration, intracellular release, and therapeutic efficacy. Rather than treating these variables as isolated engineering parameters for the conjugates, we discuss them in the context of the biological properties of each receptor, which ultimately govern conjugate uptake and intracellular processing. Readers seeking comprehensive strategies for ligand engineering, linker chemistry design, and payload selection are referred to several excellent recent reviews [1,2,3,4,5,6,7,8,9].

Finally, we consider a central question underlying TDD: to what extent do targeting ligands increase the amount of therapeutic payload that ultimately reaches cancer cells relative to administration of the free drug? While TDD approaches aim to improve tumor selectivity and reduce systemic toxicity by concentrating payloads within tumor tissue, conjugate-based therapies, including ADCs and PDCs, can still produce significant adverse effects. We therefore conclude by highlighting key determinants that govern the balance between efficacy and toxicity, including receptor choice, target density, intratumoral heterogeneity, conjugate size, linker stability, and internalization efficiency. Together, these considerations illustrate how careful alignment of receptor biology with conjugate design principles can maximize drug delivery to tumors and guide the development of more effective and selective targeted cancer therapies.

## 2. Cell Surface Receptors with Clinically Approved Conjugates

Cancer CSRs are proteins on the surface of cancer cells that play a crucial role in cell communication and growth. They can bind to specific molecules, such as growth factors or hormones, triggering signals that promote cell division and survival. These receptors are often overexpressed or mutated in cancer cells, leading to uncontrolled growth and tumor formation. Understanding these receptors helps researchers develop targeted therapies that aim to block their activity and hinder cancer cell proliferation. Another major application of CSRs is to utilize these receptors for specific uptake of drugs into cancer cells using the TDD approach [12]. Besides these, the overexpression of CSRs could be used to predict cancer outcomes and inform clinical management. In the following, we summarize four representative CSRs that are overexpressed on cancer cells and have been targeted by approved conjugates in the clinic. CSRs reside at the plasma membrane in a dynamic equilibrium driven by continuous endocytosis and recycling, a balance that is often altered following conjugate binding. Conjugate binding shifts this equilibrium toward endocytosis and subsequent lysosomal degradation rather than recycling back to the cell surface. We discuss the uptake mechanisms of four targets and the target-bound conjugates, highlighting the implications for the effectiveness of the TDD approach.

### 2.1. Human Epidermal Growth Factor Receptor 2 (HER2)

**Figure 2:**
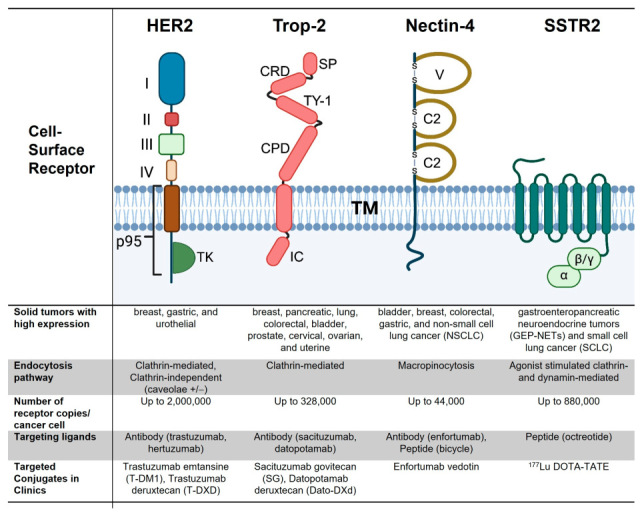
Schematic showing domain structures of four representative cell surface receptors (CSRs) targeted by FDA-approved conjugates for cancer therapy: HER2, Trop-2, Nectin-4, and SSTR2. The figure also summarizes solid tumor types with high receptor expression, receptor endocytosis pathway, approximate receptor copies per cancer cell, available targeting ligands, and FDA-approved conjugates currently in clinical use. I–IV denote extracellular domains of HER2; SP, signal peptide; CRD, cysteine-rich domain; TY-1, thyroglobulin type-1 domain; CPD, cysteine-poor domain; TM, transmembrane; IC, intracellular; V and C2 denote the V-type and C2-type domains of Nectin-4, and α, β, and γ denote G-protein subunits coupled to SSTR2.

Human epidermal growth factor receptor 2 (HER2), encoded by *ErbB2*, is a representative receptor overexpressed on the surface of cancer cells, and it has been extensively used in the development of cancer therapeutics [13]. HER2 is a transmembrane receptor protein that belongs to the ErbB family of receptor tyrosine kinases (Figure 2). Unlike other members of this family, including *ErbB1* (EGFR), *ErbB3* (HER3), and *ErbB4* (HER4), HER2 lacks a known ligand-binding domain but possesses a potent intracellular tyrosine kinase domain. HER2 plays a crucial role in regulating cell growth, survival, and differentiation. Abnormalities in HER2 expression, including gene amplification and protein overexpression, lead to dysregulated signaling pathways, such as the PI3K-AKT and MAPK pathways, which promote uncontrolled cell proliferation and tumor progression. In comparison to normal human breast tissue that has a low concentration of HER2 receptors, breast cancer cells express 40–100 fold higher expression at the cell surface resulting in 2 million CSRs/cell [14]. This large tumor-to-normal cell ratio allows direct inhibition of HER2 using therapeutic antibodies such as trastuzumab (Herceptin) and pertuzumab (Perjeta). Additionally, the extracellular domain of the overexpressed HER2 is targeted using ligand–drug conjugates such as ADCs [13]. In addition to overexpression, mutations and truncation of HER2 are also observed. For instance, a truncated form of HER2 called p95 (Figure 2), which is missing the extracellular domain, is associated with increased HER2 signaling and resistance to HER2-targeted therapies [14,15].

HER2 is characterized by high stability at the cell surface. Limited internalization is known to occur through multiple endocytosis mechanisms, including clathrin-mediated and clathrin-independent endocytosis, which leads to rapid recycling of HER2 to the surface (Figure 2) [12]. Efficient internalization typically requires receptor dimerization, e.g., homodimerization after antibody-induced crosslinking [16]. Furthermore, it was shown that the overexpression of EGFR reduced HER2 homodimerization and HER2/ADC internalization, while EGFR mAbs restored HER2/ADC endosomal trafficking [16]. Another study identified abnormal HER2 internalization as a contributing mechanism of resistance to ADCs in HER2-positive breast cancer [17]. Deficiency of SNX10, a protein that mediates intracellular trafficking, decreased HER2 recycling to the cell surface and promoted HER2 trafficking into lysosomes, contributing to decreased HER2 on cell surface and leading to ADC resistance.

**Figure 3:**
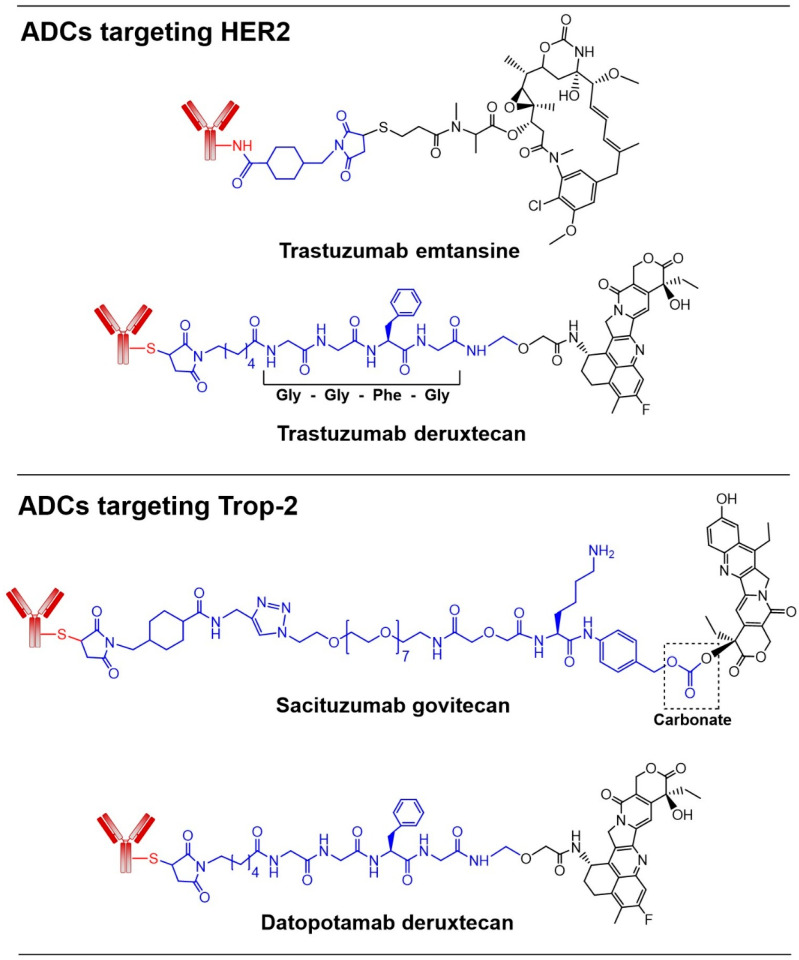
Clinically approved conjugates targeting HER2 and Trop-2. Structures of ADCs targeting HER2 or Trop-2 for uptake by cancer cells. Antibody, linker, and the drug are shown in red, blue, and black, respectively.

HER2 overexpression is predominantly observed in epithelial-origin malignancies, e.g., breast, gastric, and urothelial cancers, and is notably rare or absent in mesenchymal, neuroendocrine, CNS (brain), and kidney cancers [13]. Two ADCs that target overexpressed HER2 in cancer cells have been approved for clinical use (Figure 3). Trastuzumab emtansine (T-DM1) was the first ADC approved for advanced-stage HER2-positive breast cancer in 2013. It delivers the cytotoxic anti-microtubule agent, emtansine, to HER2-positive cancer cells via the trastuzumab antibody. However, acquired resistance to this ADC has been observed in clinical settings, likely due to drug transport polymorphisms that increase efflux and reduce intracellular drug accumulation, thereby diminishing efficacy [18]. A second HER2-targeting ADC, trastuzumab deruxtecan (T-DXD), was approved in 2019. In this conjugate, trastuzumab is linked to a topoisomerase I inhibitor via a tumor-selective enzyme cleavable linker, tetrapeptide GGFG [2]. Clinical studies have shown that T-DXD is associated with a lower risk of disease progression or death compared to T-DM1, although it also carries a higher incidence of drug-related adverse events [19]. T-DXD is approved for the treatment of previously treated advanced-stage HER2-overexpressing solid tumors (IHC 3+), regardless of tumor type, reflecting the broad prevalence of HER2 overexpression across multiple malignancies. Furthermore, third-generation ADCs targeting HER2, such as disitamab vedotin (RC48) [20], trastuzumab duocarmazine (SYD985) [21], or A166 [22], are being developed to obtain a better safety profile and positive response in patients.

### 2.2. Trophoblast Cell Surface Antigen 2 (Trop-2)

Trophoblast cell surface antigen 2 (Trop-2), coded by tumor-associated calcium signal transducer 2 (*Tacstd2*) gene, is a 46 kDa transmembrane glycoprotein, formerly detected in human trophoblast cells [23]. It is abundantly expressed in many cancers, and its overexpression has been associated with the increase in tumor growth, proliferation, and metastasis [24]. The analysis of cell surface expression of Trop-2 in six human breast cancer cell lines revealed expression levels ranging from around 32,000 to 328,000 copies per cell, with five of the six cell lines exhibiting more than 90,000 copies per cell [25]. Trop-2 is a type I membrane protein with an extracellular N-terminal domain that consists of a hydrophobic leader peptide (1–26 aa, signal peptide) and an extracellular domain (27–274 aa) (Figure 2). The N-terminal domain is attached to a single transmembrane helix (275–297 aa) followed by a short intracellular C-terminal tail (298–323 aa) that is involved in signaling [24]. The C-terminal contains the highly conserved phosphatidylinositol 4,5-biphosphate (PIP2) binding sequence, as well as the phosphorylation sites. Regulated by many transcription factors and ligands, Trop-2 initiates a signal transduction cascade when activated, ultimately leading to activation of signaling pathways such as: JAK/STAT, ERK/MAPK, and AKT, all of which are involved in cell proliferation, survival, and invasion [23]. Therefore, Trop-2 overexpression is associated with decreased patient survival, increased tumor aggressiveness, and greater metastasis in cancer patients. Overall, overexpressed Trop-2 in cancers represents an excellent target for developing various anti-cancer therapeutic strategies, with the ADC approach leading thus far. Trop-2 undergoes constitutive internalization through endocytic pathways, predominantly via clathrin-mediated endocytosis [12]. Following antibody or ADC binding, receptor crosslinking enhances Trop-2 clustering and accelerates internalization into early endosomes. From there, Trop-2 and Trop-2/ADC complexes are trafficked through the endo-lysosomal pathway, where lysosomal degradation enables intracellular payload release. While a fraction of internalized receptor may recycle back to the plasma membrane, ADC engagement typically shifts trafficking toward lysosomal sorting, thereby increasing intracellular drug delivery efficiency in Trop-2-expressing cancer cells [12].

High levels of Trop-2 have been observed in numerous cancers, including breast, cervical, colorectal, esophageal (specific subtypes), lung (certain subtypes), and oral squamous cell carcinoma [23]. Trop-2 is also prevalent in other solid tumors, such as ovarian, pancreatic, prostate, bladder, and uterine cancers, and is expressed in hematologic malignancies like non-Hodgkin’s lymphoma, chronic lymphocytic leukemia, and Raji Burkitt lymphoma. Despite its widespread overexpression in many cancers, Trop-2 is not upregulated in the majority of esophageal, head and neck, and lung tumors, indicating that its expression varies based on tumor subtypes. Furthermore, Trop-2 is notably absent in anaplastic large cell lymphoma (ALCL).

Currently, there is limited evidence supporting the efficacy of therapeutic mAbs that target Trop-2 [26]. To date, most successful applications have leveraged Trop-2-targeting antibodies as part of conjugated therapies, such as ADCs, radioimmunotherapy, photoimmunotherapy, or CAR T-cell therapies [27,28,29,30,31,32]. Notably, two Trop-2-directed ADCs have received approval for clinical use (Figure 3). Sacituzumab govitecan (SG), approved in 2021 for the treatment of metastatic triple-negative breast cancer (TNBC) patients, comprises a humanized antiTrop-22 IgG1 mAb conjugated to the DNA topoisomerase I inhibitor SN-38 (drug-to-antibody ratio or DAR of 8) via a PEG-based, acid-sensitive (carbonate) hydrolysable linker [29,30]. SN-38 is normally administered as irinotecan, the prodrug, and when converted to its active form, SN-38, it has limited bioavailability. Therefore, when SG was administered by injection, it delivered 20- to 136-fold more of the toxic payload (SN-38) to Trop-2 overexpressing tumor cells than injected irinotecan [31]. SG also lowered intestinal uptake compared to irinotecan, limiting toxic effects in the gut. Furthermore, due to the cleavable mechanism of the ADC, the cytotoxic payload SN-38 (which is membrane-permeable) is released into the tumor microenvironment, contributing to the bystander effect on the neighboring cells [30]. With the promising initial clinical outcomes and widespread overexpression of Trop-2 in several cancers, SG plus pembrolizumab is being evaluated for first-line treatment of untreated, advanced TNBC [33].

Datopotamab deruxtecan (Dato-DXd), another Trop-2-directed ADC (approved in 2025), is indicated for the treatment of metastatic breast cancer and non-small-cell lung cancer (NSCLC) [27,28]. In Dato-DXd, antiTrop-2-2 mAb is conjugated to DXd with a cleavable tetrapeptide GGFG linker (Figure 3). The cytotoxic payload DXd is 10× more potent than SN-38, and the DAR is ~4 in this ADC, as opposed to 8 in SG. The tetrapeptide linker is stable in plasma and is cleaved by lysosomal proteases after ADC uptake followed by release of the cytotoxic drug. This corresponds to a decrease in incidence of side effects such as neutropenia and diarrhea. This ADC has a wide therapeutic window that allows for high doses if needed to achieve therapeutic results. Key factors that may influence the clinical success of Dato-DXd, an ADC containing a topoisomerase I inhibitor, include topoisomerase I expression, active DNA replication and transcription, homologous recombination deficiency, and the expression of genes involved in DNA damage repair. Ongoing studies are exploring the resistance mechanisms associated with these factors and strategies to overcome them [34]. Overall, Trop-2 as a target for ADCs has piqued the interest of several companies, leading to the evaluation of multiple anti-Trop2 ADCs in clinical studies, such as sacituzumab tirumotecan [35].

### 2.3. Nectin-4

Nectin-4, also known as poliovirus receptor-like protein 4 (PVRL4), is a clinically validated target in oncology. It shows widespread expression and frequent amplification across multiple solid tumors [36]. It is one of the four transmembrane proteins in the nectin family [36,37]. Nectin-4 is a 66 kDa cell adhesion molecule composed of an extracellular domain, a transmembrane region, and a cytoplasmic tail (Figure 2) [38]. While nectin subtypes 1–3 are commonly expressed in healthy adult tissues, Nectin-4 expression is typically restricted to embryonic and placental tissues. In adults, its expression is rare and is most often observed in cancerous tissues. Notably, the detection of soluble Nectin-4 in supernatants has emerged as a potential serum biomarker for certain types of cancer. Although the precise role of Nectin-4 in tumor development and progression remains unclear, studies suggest its involvement in tumor cell motility, invasion, angiogenesis, and activation of the PI3K/AKT signaling pathway. The mechanism of Nectin-4 internalization, either alone or in complex with mAbs/ADCs, is thought to involve macropinocytosis, based on limited available evidence [12,39]. This is inferred from studies showing measles virus internalization in breast cancer (MCF7 and HTB-20) and colorectal cancer (DLD-1) cells [12], as well as studies examining ADC uptake in corneal epithelial cells [39]. The latter investigations were conducted to elucidate the mechanisms underlying ocular toxicity associated with the clinically approved Nectin-4-targeted ADC enfortumab vedotin.

The higher expression levels of Nectin-4 in several cancers, including bladder, breast, colorectal, gastric, and NSCLC, compared to normal tissues, make it a prime candidate for the development of targeted therapies [36]. Studies across 12 distinct solid tumor types found that Nectin-4 was expressed in over 50% of patients in 11 of the 12 cancer types [36]. Several human urothelial carcinoma cells lines, such as T24, RT112, UC9, UC14, and HT1376, displayed different cell surface density for Nectin-4, ranging up to about 44,000 per cell [40].

**Figure 4:**
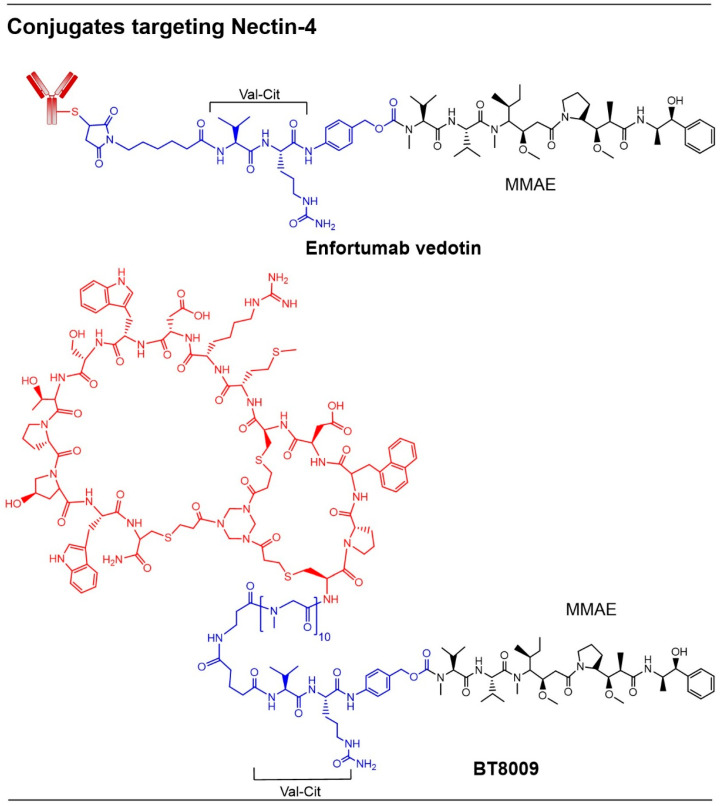
Conjugates targeting Nectin-4. Structures of Nectin-4-targeted ADC enfortumab vedotin and PDC BT8009. Antibody/peptide, linker, and the drug are shown in red, blue, and black, respectively.

Enfortumab vedotin (Figure 4), targeting Nectin-4, was approved in 2021 for adults with locally advanced or metastatic urothelial cancer who have previously received PD-1 or PD-L1 inhibitors [41]. A fully human mAb is conjugated to monomethyl auristatin E (MMAE) in this ADC, which has reduced the risk of death by ~30% with a mild to moderate safety profile compared with standard chemotherapy. Due to the cleavable linker (Val-Cit), there are several bystander effects to be observed, resulting in off-target killing of other cells.

Other conjugates, such as BT8009 (Figure 4), in development stages, utilize a bicycle peptide to target Nectin-4 [42]. The bicycle peptide exhibits high selectivity for Nectin-4 binding and incorporates unnatural amino acids that form two large rings, imparting improved physicochemical properties. The bicycle peptide is conjugated to the cytotoxin MMAE via a cleavable Val-Cit linker. Bicycle toxin conjugates represent a new class of anti-cancer agents that provide therapeutic benefit due to their small size, which allows for rapid tissue distribution and effective tumor penetration for sufficient delivery of the cytotoxic payload. The constrained bicycle conformation maintains the peptide in its preferred active structure, thereby enhancing binding to Nectin-4. A 10-unit polysarcosine spacer is incorporated to reduce potential steric clashes that could otherwise impair receptor binding. Additional applications of Nectin-4 targeting include the development of anti-Nectin-4 mAb-based constructs for immune single-photon emission computed tomography (SPECT) imaging and photothermal therapy in TNBC [43].

### 2.4. Somatostatin Receptor (SSTR)

Somatostatin receptors (SSTRs) are G-protein-coupled receptors that are expressed throughout the body and are overexpressed in many cancers [44]. Structurally, they comprise seven transmembrane segments with an extracellular N-terminal and intracellular C-terminal. Five SSTR subtypes (SSTR1–5) have been identified, serving as both diagnostic markers and therapeutic targets in various cancers. SSTRs are activated by an endogenous peptide hormone, somatostatin (SST), or its synthetic analog octreotide. Octreotide is a metabolically stable, long-acting SSTR2 agonist that induces receptor phosphorylation and internalization [45]. It is an approved treatment for acromegaly and diarrhea associated with carcinoid tumors.

SSTR2 is highly expressed in neuroendocrine tumors (NETs), which are cancers that develop in specialized cells called neuroendocrine cells [44]. Although NETs are considered rare, neuroendocrine cells are present in nearly every organ, meaning NETs can occur anywhere in the body. An overexpression of SSTR2 is most established in gastroenteropancreatic neuroendocrine tumors (GEP-NETs) and SCLC (Figure 2). In addition, SSTR2 is also overexpressed in a number of non-NETs, including breast cancer, prostate cancer, melanoma, lung cancer, gastrointestinal cancers, and gynecological cancers like ovarian and endometrial cancers [44]. Cell surface expression of SSTR2 in several medulloblastoma cell lines suggests that receptor density can be as high as 8.8 × 105 receptor copies per cell [46]. SSTR2 is internalized via clathrin- and dynamin-mediated endocytosis after agonist (ligand) binding [47]. Endogenous agonists like SST14 lead to receptor endocytosis and recycling to the plasma membrane, while peptide analogs like octreotide and lanreotide are more effective at stimulating SSTR2 endocytosis and do not recycle SSTR2 to the surface [47]. Accordingly, Kuan et al. found that mAbs bind to SSTR2 on the cell surface in medulloblastoma and glioma cells but show no significant internalization of the mAb/SSTR2 complex in the absence of the agonist [46].

**Figure 5:**
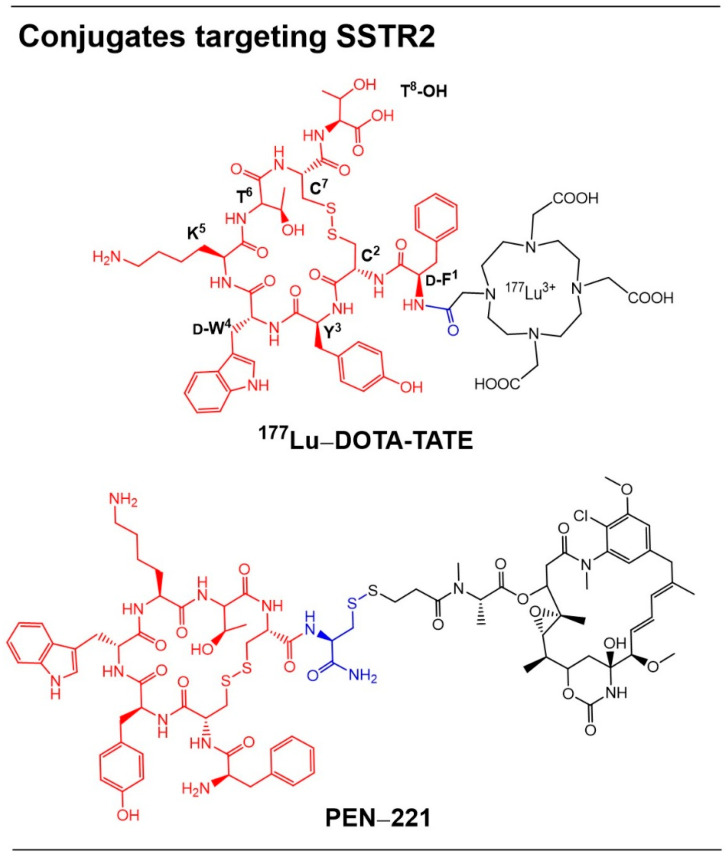
Conjugates targeting SSTR2. Structures of peptide conjugates for somatostatin receptor-targeted delivery into cancer cells. Peptide, linker, and the drug are shown in red, blue, and black, respectively.

NETs frequently arise in the gastrointestinal tract, and although surgical resection of primary and metastatic lesions remains the standard curative approach, many patients present with advanced disease and are no longer candidates for surgery [48]. This unmet clinical need drove the development of targeted therapies for GEP-NETs, including peptide receptor radionuclide therapy (PRRT). Among these, ^177^Lu DOTA-TATE (Figure 5), became the first SSTR2-targeted therapeutic approved for clinical use in 2018 [6]. It consists of peptide octreotide [(D-Phe^1^,Tyr^3^,Thr^8^)-octreotate, or TATE)], which binds selectively and with high affinity to SSTR2 on cancer cells. The peptide’s N-terminal is conjugated to a DOTA chelator carrying lutetium-177, enabling targeted delivery to tumor sites where the radionuclide emits low- to intermediate-energy b-particles [45]. This mechanism concentrates radiation within SSTR2-expressing tumors while minimizing exposure to surrounding tissues. The peptide–radionuclide conjugate is used clinically to treat metastatic progressive GEP-NETs overexpressing SSTR2, highlighting the therapeutic potential of peptide-based delivery systems in oncology [48].

New treatment strategies are also warranted for SCLC, an aggressive NET with a high mortality rate (~95%). PEN-221, a small PDC (unlike larger ADCs), was designed to overcome the challenges of SCLC biology and the efficacy limitations of ADCs and radionuclide therapies such as ^177^Lu DOTA-TATE [49]. For instance, dosing of 177Lu DOTA-TATE once every 2 months was considered too infrequent for complete regression of SCLC tumors. PEN-221 links the C-terminal side chain of octreotide to cytotoxic agent DM1 (Figure 5), and it was developed for treatment of SCLC. Treatment with PEN-221 in SCLC xenograft mouse models resulted in complete regression of tumors [50]. The small size of PEN-221 facilitated deep tumor penetration and limited plasma exposure, allowing for repeated dosing and sustained accumulation of the DM1 payload in tumor tissue (up to 5 days), resulting in durable tumor control. The conjugate was also evaluated in patients (Phase 2 study) with SSTR2-expressing advanced NETs, where it was well tolerated with a high clinical efficacy and a median progression-free survival of 9 months [51].

Another application of SSTR targeting was recently shown by the development of a theranostic ^68^Ga-MMC(TMZ)-TOC, which combines a clinically used radiotracer ^68^Ga-DOTA-TOC (where TOC stands for Tyr^3^-octreotide) with a DNA alkylating agent temozolomide (TMZ) [52]. The peptide octreotide present in this PDC ensures targeted delivery of TMZ and ^68^Ga to SSTR2-expressing tumor cells. Mice bearing both HCT116-WT and HCT116-SSTR2 xenografts (dually implanted) showed selective uptake of PDC in HCT116-SSTR2 tumors (>15-fold higher) compared to no/minimal PDC detected in WT tumors [52].

## 3. Cell Surface Receptors with Conjugates in the Development Stages

In addition to the clinically validated targets (Table 1), a number of other overexpressed CSRs are being studied for the development of drug conjugates targeting difficult-to-treat and aggressive cancers. In the following, we highlight three representative CSRs for which targeted conjugates are at various stages of clinical or preclinical development and examine their respective therapeutic opportunities and challenges.

### 3.1. Epidermal Growth Factor Receptor (EGFR)

Epidermal growth factor receptor (EGFR), a member of the ErbB family, is also often overexpressed or mutated in various cancers [53]. EGFR is composed of a single-chain transmembrane protein that encompasses an extracellular N-terminal EGF-binding domain, a tyrosine kinase domain in the cytoplasmic area, and a C-terminal phosphorylation domain. Upon binding of its ligands to the extracellular N-terminal domain, the ligand-induced activation leads to autophosphorylation of tyrosine residues present within the C-terminal domain. This activates downstream signaling pathways involved in cell proliferation, survival, and migration, including the PI3K-AKT and MAPK pathways. Mutant EGFR resulting from oncogenic alterations disrupts normal receptor endocytosis, leading to enhanced signaling activity [53]. Notably, EGFR exhibits overexpression or activating mutations in over 30% of breast cancers, approximately 60% of non-small-cell lung cancers, and around 40% of glioblastomas [54]. Normal cells express 40,000 to 100,000 receptors per cell, while there are more than 1,000,000 receptors per cancer cell.

Dysregulated EGFR signaling contributes to uncontrolled cell proliferation and tumor progression, therefore specific EGFR inhibition is one of the key strategies in cancer therapy. Numerous EGFR blockers have been developed for the treatment of EGFR-overexpressing cancers, including anti-EGFR antibodies such as cetuximab (approved in 2004) and panitumumab (approved in 2006). These therapeutic antibodies bind to the extracellular N-terminal domain of EGFR, thereby preventing receptor dimerization and subsequent activation of downstream signaling pathways. However, clinical trials with antibodies and/or tyrosine kinase inhibitors against EGFR have shown limited efficacy in several aggressive cancers, including TNBC, warranting other strategies for treatment [55]. Ligand binding to EGFR induces EGFR endocytosis and is utilized for targeted receptor-mediated uptake of drug conjugates, such as ADCs or PDCs [55,56,57,58]. The conjugate approach takes advantage of the overexpression of the target cell surface protein in cancer cells while not relying on downstream signaling.

**Figure 6:**
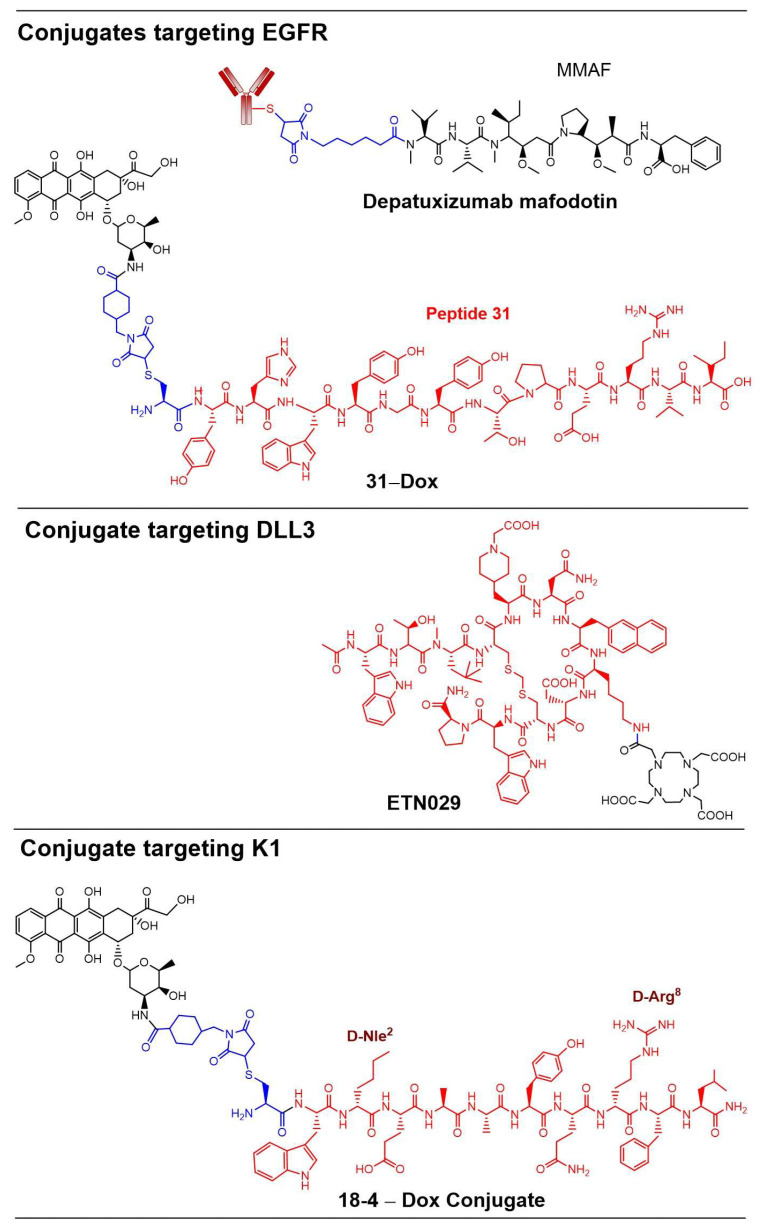
Conjugates targeting EGFR, DLL3 or K1. Structures of conjugates targeting different CSRs with an antibody or a peptide. Antibody/peptide, linker, and the drug are shown in red, blue, and black, respectively.

Several EGFR-targeted conjugates are at development stages, such as serclutamab talirine (ABBV-321) [56], depatuxizumab mafodotin (Depatux-M or ABT-414) [57,59], and 31-Dox [58]. Depatux-M (Figure 6) consists of a tumor-specific antibody, ABT-806, conjugated to monomethyl auristatin F (MMAF), a potent microtubule inhibitor, via a non-cleavable maleimidocaproyl (mc) linker [57]. After ADC internalization, the antibody is degraded in the lysosomes to release Cys-mc-MMAF. The treatment with ADC led to significant tumor growth inhibition (87 to 96%) and sustained tumor regressions in glioblastoma multiforme (GBM) patient-derived xenograft (PDX) mouse models expressing either wild-type EGFR or EGFR with mutations (EGFR variant III) [57]. Depatux-M was also compared to another EGFR-targeted ADC losatuxizumab vedotin (ABBV-221), where antibody is attached to MMAE (a cell-permeable cytotoxic agent) via a cathepsin-B cleavable linker (Val-Cit) [59]. Evaluation in three GBM PDX mouse models after direct infusion by convection-enhanced delivery (CED) showed that both ADCs (Depatux-M and ABBV-221) had comparable efficacy. However, brains infused with ABBV-221 led to profound neuronal toxicity and microglia/macrophage infiltration due to the linker instability of the ADC and cell-permeable MMAE. A bispecific ADC was also reported to target both HER2 and EGFR in cancer cells. The conjugate showed significant antitumor activity in SK-OV-3 and A-431 xenograft models that were unresponsive to HER2- or EGFR-targeted ADCs [60]. In addition, we developed an EGFR-targeted PDC, 31-Dox (Figure 6), designed for the selective delivery of the anthracycline doxorubicin (Dox) to TNBC cells. In this PDC, peptide 31, engineered to bind EGFR on TNBC cells, is conjugated to Dox via a non-cleavable succinimidyl thioether linker. Dox is clinically used as an adjuvant or neoadjuvant therapy for several cancers, including breast cancer. 31-Dox displayed selective toxicity toward TNBC cells (IC_50_ 2.3–2.7 μM) and no detectable toxicity toward normal mammalian epithelial cells even at a high concentration (25 μM) [58].

### 3.2. Delta-like Ligand 3 (Dll3)

Delta-like ligand 3 (DLL3) is an inhibitory ligand of the Notch signaling pathway and is highly upregulated and aberrantly localized to the cell surface in SCLC and other high-grade NETs [61]. The Notch pathway is a highly conserved cell–cell signaling pathway that regulates numerous cellular processes, including the differentiation of pulmonary neuroendocrine cells. In SCLC, dysregulated Notch signaling contributes to several oncogenic cellular processes, such as enhanced cell proliferation, altered differentiation, neuroendocrine plasticity, and acquisition of chemoresistance. DLL3 itself has been implicated in promoting the metastatic and treatment-resistant characteristics of NECs. High DLL3 expression is associated with advanced disease stage and poor clinical outcomes. Importantly, its cell surface expression profile has enabled the development of therapeutics designed to specifically target DLL3-expressing cancer cells.

Rovalpituzumab tesirine (or Rova-T) represented one of the earliest attempts to exploit overexpression of DLL3 in SCLC. Rova-T consists of a DLL3-specific humanized mAb (SC16) conjugated to a membrane-permeable pyrrolobenzodiazepine (PBD) dimer toxin via a cleavable Val-Ala linker [61]. Preclinical studies demonstrated superior antitumor activity compared with cisplatin in SCLC models and efficacy in patient-derived xenografts refractory to platinum-based chemotherapy, supporting its rapid translation to clinical evaluation [62]. Early clinical studies suggested that patients with DLL3-high tumors experienced higher response rates (36% survival rate) compared to patients treated with conventional chemotherapy (14%), validating DLL3 as a promising target [62]. However, subsequent trials revealed only modest clinical activity accompanied with associated adverse effects, and DLL3 expression determined by IHC did not ultimately correlate with improved overall survival. Finally, the lack of survival benefit in advanced SCLC in phase 3 studies halted further development of Rova-T [61]. Mechanistic analyses of these outcomes provided important translational insights for next-generation DLL3 ADC design. In particular, the extreme potency of the PBD payload (picomolar IC_50_) combined with the cleavable Val-Ala linker likely promoted premature payload release in circulation. Because PBD dimer is membrane-permeable, its premature release may have enabled diffusion into non-malignant tissues, producing systemic toxicities and a pronounced bystander effect, limitations that have since informed efforts to optimize linker stability, payload potency, and tumor selectivity in subsequent DLL3-targeting ADCs.

One example of this next-generation strategy is DB-1314, which was designed explicitly to address the translational challenges experienced with Rova-T [63]. DB-1314 consists of a fully humanized mAb that binds a different epitope on DLL3 than the Rova-T mAb, and it is conjugated to DXd topoisomerase I inhibitor via a maleimide peptidyl (GGFG) linker to give a DAR of 8. The DXd payload is several-fold less cytotoxic compared to the PBD dimer, reflecting a deliberate shift toward a broader therapeutic window while maintaining potent antitumor activity [9]. Consistent with this design rationale, DB-1314 exhibited potent cytotoxicity against multiple DLL3-positive SCLC cells, including those with low surface DLL3 expression (<1000 molecules per cell). In SCLC PDX mouse models with variable DLL3 expression, the ADC produced robust tumor regressions and showed antitumor activity comparable to or exceeding that of an ADC targeting B7H3 in the same models, independent of the relative expression levels of the two antigens. Importantly, DB-1314 also showed a favorable safety profile in cynomolgus monkeys, with a maximum tolerated dose greater than 60 mg/kg [63]. Collectively, these findings illustrate how lessons learned from Rova-T, particularly regarding payload potency, linker stability, and bystander toxicity, are shaping the rational design of next-generation DLL3-directed ADCs with improved translational potential. Additionally, a DLL3-targeted peptide-DOTA radioligand carrier ETN029 (Figure 6) was recently reported to have entered phase 1 trials for SCLC and neuroendocrine prostate cancer [64]. This construct employs a macrocyclic peptide with picomolar affinity to human DLL3, conjugated to a DOTA chelator, facilitating specific tumor uptake and internalization.

### 3.3. Keratin 1 (K1)

Keratin 1 (K1) is a type II intermediate filament protein that is found to be overexpressed and localized to the cell surface in certain breast cancer subtypes, including estrogen receptor-positive/progesterone receptor-positive (ER+/PR+) and TNBC [65,66]. In particular, TNBC cell lines exhibited intense peripheral staining, indicating the presence of cell surface K1 (CSK1) [66]. In contrast, non-cancerous mammary epithelial cells (MCF-10A and MCF-12A) showed little to no surface staining, suggesting minimal CSK1 expression in normal breast tissue. These findings support the potential of K1 as a therapeutic target in both TNBC and ER+/PR+ breast cancer subtypes. Of note, K1 overexpression is especially significant in TNBC, a subtype with limited targeted treatment options, while ER+/PR+ breast cancer can be treated with hormone-based therapies, and TNBC lacks such targeted interventions. Moreover, grade 3 TNBC tumors exhibit significantly higher K1 expression (>3-fold) compared to grade 2 tumors [66]. This differential expression further highlights the promise of CSK1 as a target for TNBC-specific drug delivery strategies. The expression of CSK1 in neuroblastoma NMB7 cells [67] and elevated K1 in hepatocellular carcinoma (HCC) patients have also been reported [68].

We evaluated several K1-targeted PDCs for the specific delivery of Dox to TNBC cells via CSK1-mediated endocytosis. The PDCs demonstrated selective cytotoxicity in both cell-based systems [69,70] and xenograft mouse models [71,72]. These conjugates were selectively cytotoxic to TNBC cells while exhibiting up to 30-fold reduced toxicity toward normal mammary epithelial cells. In mice models bearing orthotopic TNBC (MDA-MB-231) xenografts, four weekly injections of 18-4–Dox conjugate (Figure 6) resulted in significantly reduced tumor volumes compared to the treatment with free Dox at an equivalent dose [72]. The IHC analysis of tumor tissues revealed that the low-dose PDC treatment (2.5 mg/kg of Dox equivalent) decreased the expression of proliferation markers (PCNA and Ki-67) and increased apoptotic signaling, as indicated by elevated caspase-3 expression. In contrast, the same dose of free Dox produced marker expression levels comparable to saline-treated controls. Notably, tumors from PDC-treated mice accumulated 7.6-fold more Dox compared to those treated with free Dox, while off-target accumulation in organs such as the liver, heart, and lungs was significantly reduced (up to three-fold), suggesting enhanced tumor selectivity and reduced systemic toxicity. Building on these findings, the application of a cyclic peptide analog (cy18-4) of 18-4 for targeting K1 in aggressive cancers is currently being explored for the development of both therapeutic and imaging agents [73,74].

Overall, we focused on seven representative CSRs, four clinically validated targets and three emerging targets, to illustrate how receptor-specific properties vary and how conjugates (ADCs or PDCs) have been designed to target these for the treatment of different cancer types. Importantly, once a CSR is selected for TDD in a given cancer, the resulting conjugates differ in targeting ligand (mAb, peptide, etc.), linker chemistry, and payload, which in turn influence circulation half-life, tumor penetration, bystander effects, intratumoral drug concentration, and overall therapeutic efficacy. In the following section, we further discuss several of these key parameters and their impact on tumor drug accumulation. Besides these CSRs, a number of other receptors, including Glypican 3 [75], GRP78 [76,77], and claudin 18.2 [78], are being identified and developed for targeted drug delivery in cancer therapy. It is likely that additional CSRs with clinical candidate conjugates will emerge in the coming years.

## 4. Drug Concentration at the Tumor Site

The goal of drug (payload) delivery using a targeting ligand–receptor-mediated uptake approach is to increase drug concentration at the tumor site, thereby enhancing cellular uptake compared with administration of the free drug. Following systemic administration via the intravenous route, the conjugate or the free drug rapidly distributes throughout the body. However, the small-molecule drug is typically cleared rapidly, whereas conjugates (such as ADCs or PDCs) exhibit prolonged circulation half-life and selective uptake by tumor tissues. Notably, even for the conjugates, the majority of the administered or injected dose (ID) is lost due to catabolism within healthy cells through various pathways [3], and only a small fraction perfuses into the solid tumor. Nevertheless, it has been shown that with careful conjugate design, up to ~11% of the injected dose can be delivered to the solid tumor following systemic administration, despite only a minor fraction (<2%) of the drug entering the tumor during each circulatory cycle [79]. Also, studies indicate increasing tumor drug concentration up to several-fold—21-fold (Table 2) or higher compared to when free drug is administered—are achievable using targeting approaches. As discussed below, factors such as higher target expression per cell, improved tissue penetration and distribution, enhanced cellular uptake, and intermediate linker stability are critical determinants of tumor drug concentration and overall therapeutic efficacy.

With the conjugate approach, higher expression of the targeted CSR in cancer cells leads to higher drug levels in the tumor and enhanced efficacy. For instance, evaluation of an ADC targeting Trop-2, Dato-DXd (Figure 3), administered at 10 mg/kg in 11 breast cancer PDX mice with varying Trop-2 expression showed that higher Trop-2 expression (determined by IHC and RPPA) led to higher DXd in tumor tissues and enhanced antitumor activity [80]. The measurement of DXd concentration in tumor tissue 72 h after the administration of Dato-DXd or IgG-DXd isotype control antibody conjugate showed that Dato-DXd-treated mice had up to ~17-fold higher DXd levels compared to the mice treated with the isotype control conjugate (Table 2). Similarly, SN-38 levels in tumors were 20.8-fold higher in mice bearing NCI-N87 tumors that received SG than in those that received irinotecan [31]. In another mouse model with Capan-1 tumors, a delivery advantage of 45- to 136-fold higher was reported for SG over irinotecan [31]. Another study aimed to quantify the relationship between antigen expression levels and ADC efficacy used trastuzumab-valine-citrulline-monomethyl auristatin E (T-vc-MMAE) and four breast cancer cell lines (SKBR-3, MDA-MB-453, MCF-7, and MDA-MB-468) with varying HER2 expression levels (800,000, 250,000, 50,000, and 10,000 receptors/cell, respectively) as models [81]. After the incubation of cancer cells with T-vc-MMAE, a linear relationship between HER2 expression and intracellular free MMAE was observed. However, increasing the concentration beyond the target saturation level did not result in higher payload exposure.

For glioma-targeted drug delivery of Dox, a PDC (pHA-AOHX-VAP-DOX) was reported to increase Dox concentrations in tumor tissues across the BBB compared to the administration of free Dox [77]. In this PDC, the targeting ligand was designed to bind two receptors and facilitate delivery of the conjugated drug across the blood–brain tumor barrier. The targeting ligand consisted of a 7-mer peptide (called VAP with all D-amino acids) and p-hydroxy benzoic acid (pHA) for binding to glucose-regulated protein 78 (GRP78, a heat shock protein) and dopamine receptor (D2), respectively, in brain tumors. GRP78 is a heat shock protein known to be exclusively expressed on the surface of tumor cells while being absent from normal cells. GRP78 facilitated glioma cell uptake and internalization of the conjugate via binding to the VAP, followed by receptor-mediated endocytosis. In addition, the dopamine D2 receptor present in the central nervous system was targeted in the brain with a benzamide analog, pHA. Both the VAP peptide and pHA were conjugated to Dox via a hydrophilic linker to obtain the PDC with high aqueous solubility. In vitro data showed that the PDC was internalized into lysosomes in U87 and HUVEC cells and was cytotoxic to these cells. In mice with intracranial U87 glioma, PDC treatment led to significantly higher drug accumulation in tumors and demonstrated enhanced anti-glioma efficacy compared to mice treated with free Dox. Biodistribution showed that 2 h after PDC administration, 4.7-fold more drug was present in the tumor compared to free Dox administration (Table 2). Normal organs like the heart, liver, spleen, and lungs showed ~2-fold less drug after PDC treatment in comparison to Dox treatment.

In female NIH-III mice bearing orthotopic TNBC tumors, 24 h after intravenous administration of PDC 18-4–Dox (also called amide PDC, Figure 6), 7.6-fold higher Dox accumulated in tumors compared to free Dox-treated mice (Table 2) [72]. We also evaluated another PDC, named hydrazone PDC, where peptide 18-4 was linked to Dox via an acid-labile *N*-acyl hydrazone linker [71]. The PDC showed selective toxicity toward TNBC cells; however, it was less selective compared to the amide PDC. The hydrazone PDC exhibited a shorter half-life in human serum (t_1/2_ ~6 h) compared to the amide PDC (t_1/2_ ~18 h). Accordingly, female NOD-SCID mice bearing subcutaneous TNBC xenografts treated with the hydrazone PDC showed only a 1.4-fold increase in tumor Dox concentration relative to free Dox-treated mice, whereas amide PDC-treated mice showed a much higher tumor Dox concentration (7.6-fold) compared to the free Dox group (Table 2). These findings highlight the significance of linker stability in optimizing the pharmacokinetics and biodistribution of the drug at the tumor site. Other studies also show that conjugates with linkers of varying serum half-lives exhibit different therapeutic outcomes in mice, with those containing linkers of intermediate stability demonstrating the most effective therapeutic response [25,82]. In contrast, conjugates with highly stable linkers were significantly less effective. Furthermore, PDC displayed a longer circulation time compared to the free drug. The evaluation of the circulation time of hydrazone PDC after tail-vein injection in mice showed that PDC remained in the blood for up to 4 h. In comparison, mice injected with free Dox showed much lower circulation time (detected only at 15 min) [71].

In addition to achieving high drug concentrations at the tumor site, efficient tumor penetration and uniform intratumoral distribution are essential for optimal therapeutic efficacy, as most drugs tend to accumulate primarily near the tumor periphery or in regions adjacent to blood vessels. Smaller conjugates can diffuse more deeply into tumor tissue before binding to and being internalized by target cells. Nessler et al. compared the in vivo tissue penetration of three constructs consisting of PSMA-binding single-domain ADCs and an IgG–drug conjugate in DU145-PSMA xenografts [83]. The three smaller conjugates consisted of different fused heavy-chain domains (V_H_) with sizes of 29.7 kDa, 31.1 kDa, and 44.7 kDa. The 31.1 kDa (V_H1_-HLE-AF680) construct showed the highest tissue penetration, targeting a larger number of cells compared to the other two constructs or the IgG–AF680 conjugate (~150 kDa). For ADCs, tumor penetration can also be enhanced by co-administering an unconjugated antibody, which partially blocks CSR binding sites, thereby allowing the ADC to penetrate deeper into tumor tissue [84,85]. A phase I study involving 24 patients with head and neck squamous cell carcinomas (HNSCC) evaluated whether an unconjugated antibody panitumumab could improve the microscopic distribution of panitumumab-IRDye800CW in a clinical setting [84]. The analysis of excised tumor tissues revealed that the administration of the antibody–dye conjugate alone resulted in extravasation from blood vessels, followed by the diffusion through the extracellular matrix and saturation of EGFR in the outer layers of tumor nests. In contrast, co-administration with the parent (unconjugated) antibody partially occupied binding sites in these outer layers, allowing the conjugated antibody to penetrate deeper into the interior of the tumor nests and achieve a more uniform intratumoral distribution.

In addition to the strategies mentioned above, to increase drug concentration at the tumor site and improve tumor penetration, the use of optimized conjugates that permit higher dosing and longer drug residence time (e.g., by reducing efflux) in the tumor can further enhance efficacy. Moreover, local drug concentration within the tumor can be increased using multiple CSR-targeted conjugates that bind to distinct overexpressed receptors on cancer cells. Such an approach can prolong tumor residence time via multivalent binding prior to cellular internalization in tumors with a high degree of intratumoral heterogeneity. For instance, treatment combinations in which the total dose is split between SSTR2- and DLL3-targeted conjugates, rather than administered as a single receptor-targeted conjugate, could be evaluated for the treatment of NETs.

## 5. Conclusions and Future Outlook

The clinical success of CSR-targeted conjugates in delivering small-molecule chemotherapeutics to tumors has laid a strong foundation for the development of more efficacious therapeutic platforms. Nevertheless, conjugates cannot yet be considered superior to conventional chemotherapy, as traditional cytotoxic agents remain the first-line therapy for many aggressive and advanced cancers, whereas conjugates are mostly employed in second- or third-line settings. This review highlights several observations regarding the design of conjugates for improving efficacy and reducing uptake by normal tissues, which include the following:

i. High CSR expression in tumors, combined with low or no expression in normal cells and tissues, is essential for CSR-targeted conjugates in cancer therapy. Although high expression levels (for example, more than 10^6^ receptors per cancer cell) are ideal, even much lower levels (around 1000 receptors per cell) can still provide adequate payload delivery. The clinical success of T-DXD in HER2-low breast cancer [13], and the activity of conjugates targeting other CSRs with low expression, such as DLL3 (<1000 per cell) in SCLC [63], indicate that only a low threshold of CSR expression may be sufficient for effective uptake. Notably, differences among receptor quantification methods, including the IHC scoring, quantitative proteomics, and imaging-based approaches, can significantly affect receptor expression thresholds. In addition, using combinations of two or more conjugates that target different CSRs, while keeping the total dose equal to that of a single conjugate, can raise local payload concentrations and ensure uptake in all cancer cells. This strategy may be especially useful for aggressive tumors with high intratumoral heterogeneity, such as SCLC and TNBC. Furthermore, the internalization kinetics and intracellular trafficking pathways of CSR conjugate complexes are key determinants of payload delivery and therapeutic efficacy. However, the internalization mechanisms of CSRs used for TDD have not been fully characterized. Different CSRs follow distinct intracellular trafficking pathways for their bound conjugates (Figure 2). In general, receptors that recycle back to the cell surface after releasing the conjugate are preferred over those that traffic to lysosomes together with the conjugate and are subsequently degraded.
ii. Smaller conjugates can improve tumor penetration and therapeutic outcomes. Antibody fragments without the Fc region [83] and peptide [7,8,9] drug conjugates offer promising alternatives to conventional ADCs. These intermediate-size conjugates remain in circulation much longer than small molecules, which are cleared within minutes (for example, doxorubicin has a circulation half-life of 5 to 10 minutes). At the same time, they penetrate tumors more effectively because they can move through the limited spaces within tumor tissue better than full-length antibodies. The targeting component, whether a peptide or an antibody, primarily prolongs the drug’s local presence near tumor cells (residence time) rather than actively transporting it from the site of administration. By slowing rapid clearance, these components increase residence time and the probability of interaction with cellular receptors. Overall, intermediate-sized conjugates achieve an optimal balance of improved pharmacokinetics, enhanced tumor penetration, and greater tumor residence time and probability of interaction. In addition, compared with larger conjugates, intermediate-size conjugates may present fewer manufacturing and scalability challenges, including issues related to reproducible conjugation chemistry, product heterogeneity, and large-scale production.
iii. The linker chemistry plays a critical role in conjugate design, as it must maintain payload attachment during circulation to allow the conjugate to reach the tumor, while ideally releasing the drug only after internalization by cancer cells. Interestingly, linkers with very high stability often perform worse than those with intermediate stability, which retain the payload during circulation but allow rapid release at the tumor site. For example, the acid-labile carbonate linker in SG (Figure 3) showed superior in vivo activity compared with ADCs using more stable, enzyme-cleavable linkers [31,82]. The DAR of 8 increased tumor drug concentration for SG, enabled by the SN-38 payload, whereas ultra-toxic agents such as PBD dimer require lower DARs. Similarly, an amide linker in an amide PDC produced higher tumor drug levels than an extremely acid-labile N-acyl hydrazone linker in a hydrazone PDC (Table 2) [71,72]. These findings emphasize that optimal therapeutic efficacy depends on balancing linker stability in the bloodstream with efficient payload release within the tumor microenvironment.

Despite their improved selectivity, drug conjugates can still elicit substantial toxicity [33], highlighting the critical importance of optimizing both target selection and molecular design. The overexpression of CSRs remains a compelling avenue for targeted drug delivery, and next-generation conjugates, guided by emerging mechanistic insights, have the potential to maximize payload accumulation in tumors while minimizing off-target toxicities. Together, these advances are poised to enhance therapeutic efficacy and broaden clinical benefit for patients with cancer.

## Figures and Tables

**Table 1:** FDA-approved targeted ligand–drug conjugates for treatment of solid tumors.

Conjugate	Year Approved	Target CSR	Approved for Cancer Types
*Antibody-Drug Conjugates (ADCs)*			
Telisotuzumab vedotin (Emrelis)	2025	MET	NSCLC
Datopotamab deruxtecan (Datroway)	2025	Trop-2	HR+/HER2− BC
Sacituzumab govitecan (Trodelvy)	2020	Trop-2	TNBC, HR+/HER2− MBC
Mirvetuximab soravtansine (Elahere)	2022	FRα	Ovarian
Tisotumab vedotin (Tivdak)	2021	TF	Cervical
Enfortumab vedotin (Padcev)	2019	Nectin-4	Urothelial cancer
Trastuzumab deruxtecan (Enhertu)	2019	HER2	HER2+, HER2− low, NSCLC
Trastuzumab emtansine (Kadcyla)	2013	HER2	HER2+
*Peptide-Drug (Radionuclide) Conjugates (PDCs)*			
^177^Lu vipivotide tetraxetan (Pluvicto)	2022	PSMA	Prostate cancer
^177^Lu DOTA-TATE (Lutathera)	2018	SSTR2	SSTR2 +ive cancers

BC, breast cancer; CSR, cell-surface receptor; DOTA-TATE, 1,4,7,10-tetraazacyclododecane-1,4,7,10-tetracetatic acid—Tyr^3^-octreotate; FRα, folate receptor alpha; HER2, human epidermal growth factor receptor 2; HR+/HER2−, hormone receptor positive/human epidermal growth factor receptor negative; ^177^Lu, Lutetium 177; MBC, metastatic breast cancer; MET, receptor tyrosine kinase encoded by MET proto-oncogene; NSCLC, non-small-cell lung cancer; PSMA, prostate-specific membrane antigen, SSTR2, somatostatin receptor subtype 2; TF, tissue factor; Trop-2, trophoblast cell surface antigen 2.

**Table 2:** Drug concentration in tumor after administration of conjugate or free drug.

Intravenous Injection	[Drug] in Tumor Tissue	Fold Increase in [Drug] *	Reference **
Dato-DXd	12 ng/g	17	[80]
IgG-DXd	0.7 ng/g		
Sacituzumab govitecan (SG)	43.88 μg/g	20.8	[31]
Irinotecan	2.11 μg/g		
pHA-AOHX-VAP-DOX	0.68% ID/g	4.7	[77]
Dox	0.145% ID/g		
Amide PDC (Peptide 18-4—Dox)	0.219 μg/g	7.6	[72]
Dox	0.029 μg/g		
Hydrazone PDC (Peptide 18-4—Dox)	1.703 μg/g	1.4	[71]
Dox	1.205 μg/g		

* Fold increase in drug concentration in tumor tissue after conjugate administration compared to free drug or isotype control conjugate administration. ** These studies were conducted using different tumor models, dosing regimens, and sampling time points at which drug concentrations were measured after administration.

## Data Availability

No new data were created or analyzed in this study.

## Abbreviations

ADCs, antibody–drug conjugates; CSRs, cell surface receptors; DAR, drug-to-antibody ratio; DLL3, delta-like ligand 3; EGFR, epidermal growth factor receptor; GEP-NETs, gastroenteropancreatic neuroendocrine tumors; HER2, human epidermal growth factor receptor 2; ID, injected dose; IHC, immunohistochemistry; K1, keratin 1; mAb, monoclonal antibody; MMAE, monomethyl auristatin E; MMAF, monomethyl auristatin F; NETs, neuroendocrine tumors; PDB, pyrrolobenzodiazepine; PDCs, peptide–drug conjugates; PK, pharmacokinetics; PRRT, peptide receptor radionuclide therapy; PSMA, prostate specific membrane antigen; Rova-T, rovalpituzumab tesirine; SCLC, small cell lung cancer; SG, sacituzumab govitecan; SSTR, somatostatin receptor; TDD, targeted drug delivery; T-DXD, trastuzumab deruxtecan; TME, tumor microenvironment; TNBC, triple-negative breast cancer.
